# Quality Control for Medical Devices: A Review of Detection Technologies, Regulatory Standards and Matrix Interference Solutions

**DOI:** 10.3390/s26154821

**Published:** 2026-07-30

**Authors:** Xindan Zhang, Yanping Xian, Guoshan He

**Affiliations:** Guangzhou Medical Devices Testing Center, Guangzhou Quality Testing & Inspection Institute, Guangzhou 511447, China; guoyawen1@mails.gdut.edu.cn (X.Z.);

**Keywords:** lipopolysaccharide (LPS), endotoxin detection, medical devices, biosensors, microfluidics, electrochemical sensors

## Abstract

**Highlights:**

Q: What is the core focus of this systematic review?
A: This Review delivers a targeted systematic assessment of lipopolysaccharide detection technologies tailored to the distinct matrix characteristics and regulatory frameworks governing medical device quality control.
Q: Which biomimetic strategies can overcome the drawbacks of traditional pharmacopoeial endotoxin assays?
A: TLR4 pattern-recognition receptors and genetically engineered capture elements are key biomimetic recognition approaches. They effectively address the specificity limitations and false-positive issues of traditional pharmacopoeial assays.
Q: What core technologies enable fully automated and miniaturised endotoxin detection workflows?
A: EIS interfacial engineering, microfluidic system integration and intelligent data analysis are three core technologies that support fully automated, miniaturised endotoxin detection workflows.
Q: What unique contributions does this review make compared with general LPS detection reviews?
A: Unlike general LPS detection reviews, this work systematically addresses device-specific matrix interferences (LER phenomenon, silicone oil encapsulation, polymer degradation products) and regulatory frameworks, filling a critical gap in endotoxin testing literature for medical device scenarios. It also clarifies key translational barriers and research priorities for next-generation regulatory-compliant detection platforms.

**What are the main findings?**

- A systematic cross-comparison of four dominant endotoxin detection modalities demonstrates that TLR4-mediated biomimetic recognition and genetically engineered capture elements substantially surmount the specificity limitations and false-positive artefacts of conventional pharmacopoeial assays in complex medical device extract matrices.
- The synergistic integration of electrochemical impedance spectroscopy, interfacial engineering, microfluidic system architectures and intelligent signal analysis forms the foundational technical framework for fully automated endotoxin detection workflows, while methodological standardisation and regulatory equivalence validation represent the primary translational barriers to industrial-scale deployment.

**What are the implications of the main findings?**

- These findings reorient research priorities for medical device endotoxin detection: the core bottlenecks are matrix compatibility and regulatory fitness, not raw analytical sensitivity.
- This integrated technical strategy offers a potential pathway to shift endotoxin quality control from offline laboratory assays to inline real-time monitoring, with important implications for global pharmacopoeial harmonisation and industrial quality advancement.

**Abstract:**

Lipopolysaccharide (LPS), as the core active component of endotoxins from Gram-negative bacteria, serves as a mandatory quality control indicator in the biological safety evaluation of blood- and body fluid-contacting medical devices. Its rapid and accurate detection is of paramount importance for ensuring clinical patient safety and meeting global regulatory compliance requirements. Traditional endotoxin detection methods have inherent limitations, including poor anti-interference capability against medical device-specific matrices, limited automation capacity, and narrow regulatory applicability, which hinder their utility in meeting the practical demands of the modern medical device industry for high-throughput online quality control and on-site rapid screening. This review focuses on medical device-specific regulatory requirements, characteristics of extract matrices, and full-process quality control scenarios, and systematically summarizes the research progress in novel LPS-targeted biosensing detection technologies. By constructing a three-layer technical framework (biological recognition, interface engineering, and system integration), we provide a comparative assessment of four mainstream technologies—TLR4 biomimetic sensing, genetically engineered elements, EIS, and microfluidics—based on their matrix compatibility, automation potential, and regulatory compliance. It deeply analyzes the core role of microfluidic integration technology in driving the upgrading of detection systems towards automation, miniaturization and compliance, and objectively evaluates the applicability and limitations of various technologies in medical device scenarios. Unlike general reviews on LPS detection, this work specifically addresses endotoxin testing within the medical device quality control context. We systematically examine device-specific matrix interferences and regulatory landscapes, synthesizing cutting-edge research with critical analysis of industrialization bottlenecks. This review provides a theoretical foundation and technical roadmap for developing next-generation, pharmacopeial-compliant rapid automated endotoxin detection platforms—accelerating the advancement of medical device safety standards.

## 1. Introduction

Endotoxin contamination is the core biosafety risk faced by medical devices that come into direct or indirect contact with blood and body fluids, such as implants, catheters, and dialyzers [1]. LPS is the main constituent of the outer membrane of Gram-negative bacteria and the key substance responsible for the biological activity of endotoxins [2,3]. Released during bacterial proliferation or lysis, LPS can contaminate medical devices and subsequently enter the bloodstream via implantation or infusion. This activates the innate immune system, precipitating life-threatening conditions ranging from fever and septic shock to death [2,4]. Therefore, establishing reliable, high-sensitivity, and specific assays for detecting LPS on device surfaces and in extracts is imperative for quality assurance across the entire lifecycle—from R&D and production to clinical utilization [2].

Endotoxin safety risks are strictly regulated globally. An analysis of global medical device recall data from 2008 to 2021 indicates that 95% of endotoxin-related recalls involved medical devices, predominantly driven by false-negative results due to lapses in production contamination control and limited anti-interference capabilities of existing detection methods [5]. Consequently, the establishment of scientific detection standards and limit requirements is paramount throughout the production and use cycle. Globally harmonized regulations, such as ISO 10993-12 [6], GB/T 14233.2 [7], USP <85> [8], and EP 2.6.14 [9], mandate specific endotoxin thresholds based on contact type. Notably, limits are set at 2.15 EU/device for cerebrospinal fluid-contacting devices and 20 EU/device for cardiovascular implants [1,10]. The Limulus Amebocyte Lysate (LAL) assay is the current pharmacopoeial gold standard for endotoxin detection [11,12]. This method relies on the enzymatic cascade reaction within the lysate—centered on Factor C activation—to specifically detect LPS, achieving ultra-high sensitivity at the picogram (pg) level [11,13]. However, the LAL assay exhibits significant inherent limitations in medical device quality control. Its heavy reliance on the blood of endangered horseshoe crabs raises serious concerns regarding ecological conservation and animal ethics [14]. Furthermore, the global supply of reagents is perpetually constrained by the finite nature of these biological resources [15,16]. However, the LAL assay is highly susceptible to interference from medical device-specific matrices. (1→3)-β-D-glucan present in samples can inadvertently activate the Factor G pathway, leading to false-positive results [17]. Conversely, constituents such as catheter lubricants, polymeric degradation products, and sterilization by-products are prone to masking or binding endotoxins, thereby inducing the Low Endotoxin Recovery (LER) phenomenon and yielding false-negative outcomes [18,19]. Moreover, the LAL assay is operationally labor-intensive and time-consuming, typically requiring several hours to complete the sequential steps of sample pretreatment, incubation, and result interpretation [20]. This temporal demand precludes its utility in time-sensitive scenarios, such as inline cleaning validation on medical device production lines and rapid pre-implantation screening. Finally, the assay is inherently difficult to fully automate, exhibiting high dependence on manual handling and substantial inter-batch variability [21]. Consequently, it fails to satisfy the high-throughput and standardized quality control prerequisites of modern medical device manufacturing [22].

To overcome the operational bottlenecks of legacy methods, the field has shifted toward innovative solutions such as the recombinant Factor C (rFC) assay [23]. This sustainable approach replaces the need for horseshoe crab blood by employing recombinant C-factor [24]. It effectively removes confounding matrix components, offering markedly improved specificity and batch stability over conventional LAL testing [25,26]. Parallel advancements involve the fusion of biotech, nanotech, and microprocessing, which has given rise to biosensors with new recognition and signaling mechanisms for medical device LPS detection [27]. Utilizing specific capture elements (e.g., TLR4/MD-2, engineered antibodies, aptamers) alongside diverse sensing modes (electrochemical, optical, piezoelectric), these technologies markedly improve interference resistance and automation compatibility [28].

Notably, the cross-disciplinary integration of microfluidics and novel biosensing technologies is driving endotoxin detection for medical devices towards greater integration, automation and miniaturization [22,29]. However, existing reviews often focus on the biochemical mechanisms of LPS recognition or the pursuit of ultra-low detection limits in ideal buffers, neglecting the complex reality of medical device manufacturing environments. Therefore, the primary novelty of this work lies in its specific focus on the “industrial translation” of these technologies. We systematically examine device-specific matrix interferences (such as the LER phenomenon induced by silicone oil and polymers) and regulatory landscapes, synthesizing cutting-edge research with critical analysis of industrialization bottlenecks. This review focuses on the specific requirements of medical device scenarios, systematically reviews the innovative progress, scenario applicability and industrialization bottlenecks of various novel detection technologies, and provides a valuable reference for the development of the next-generation compliant and automated endotoxin detection platforms.

A systematic literature search was conducted to identify relevant studies on endotoxin (LPS) detection and medical device quality control. Four major databases were queried: Web of Science Core Collection, PubMed, Scopus, and Google Scholar. The search strategy employed the following keywords: “Endotoxin detection,” “low endotoxin recovery,” “LPS biosensor,” “Medical device quality control,” “TLR4 biosensor,” and “Microfluidics.” Publications from 2018 to 2026 were primarily included to capture the most recent technological advances and the latest updates in regulatory standards and pharmacopoeia requirements governing medical device quality control. In addition, a selection of seminal foundational works—such as the establishment of the Limulus Amebocyte Lysate (LAL) assay and structural elucidation of the TLR4/MD-2 complex—were incorporated to provide necessary background context.

## 2. Unique Challenges of Endotoxin Detection for Medical Devices

### 2.1. Device-Specific Regulatory Framework for Endotoxin Testing in Medical Devices

Regulatory standards stipulate differentiated endotoxin limit thresholds based on medical device risk classifications. Implantable devices in direct contact with blood or cerebrospinal fluid are subject to the most stringent limits (typically ≤0.5 EU/mL of extract), whereas non-invasive devices contacting mucous membranes are permitted relatively higher limits. Crucially, all detection methods must pass rigorous interference tests, demonstrating that the spike recovery rate of LPS within the specific medical device extract matrix falls within the 50–200% acceptance range specified by pharmacopoeias. This requirement represents the most fundamental application bottleneck for conventional detection methods in medical device scenarios. The core regulatory requirements of major global standards for medical device endotoxin detection are summarized in Table 1.

### 2.2. Scenario-Specific Limitations of Traditional Methods in Medical Device Quality Control

Traditional LAL and rFC assays face two fundamental limitations in medical device quality control: unreliable results caused by matrix interference and mismatch between process characteristics and application scenarios [31]. Among them, the LER phenomenon induced by medical device matrices is the most prominent industry-wide problem [32]. Medical device extracts often exhibit substantially different matrix characteristics from those of typical pharmaceuticals [33]. They often contain high concentrations of silicone oil lubricants, polymer degradation products (lactic acid produced by the hydrolysis of polylactic acid, PLA), ethylene oxide sterilization by-products, plasticizers such as di(2-ethylhexyl) phthalate (DEHP), and other components [34,35]. These substances can interfere with detection through multiple mechanisms. The core interfering substances and their mechanisms of action are shown in Table 2.

**Figure 1:**
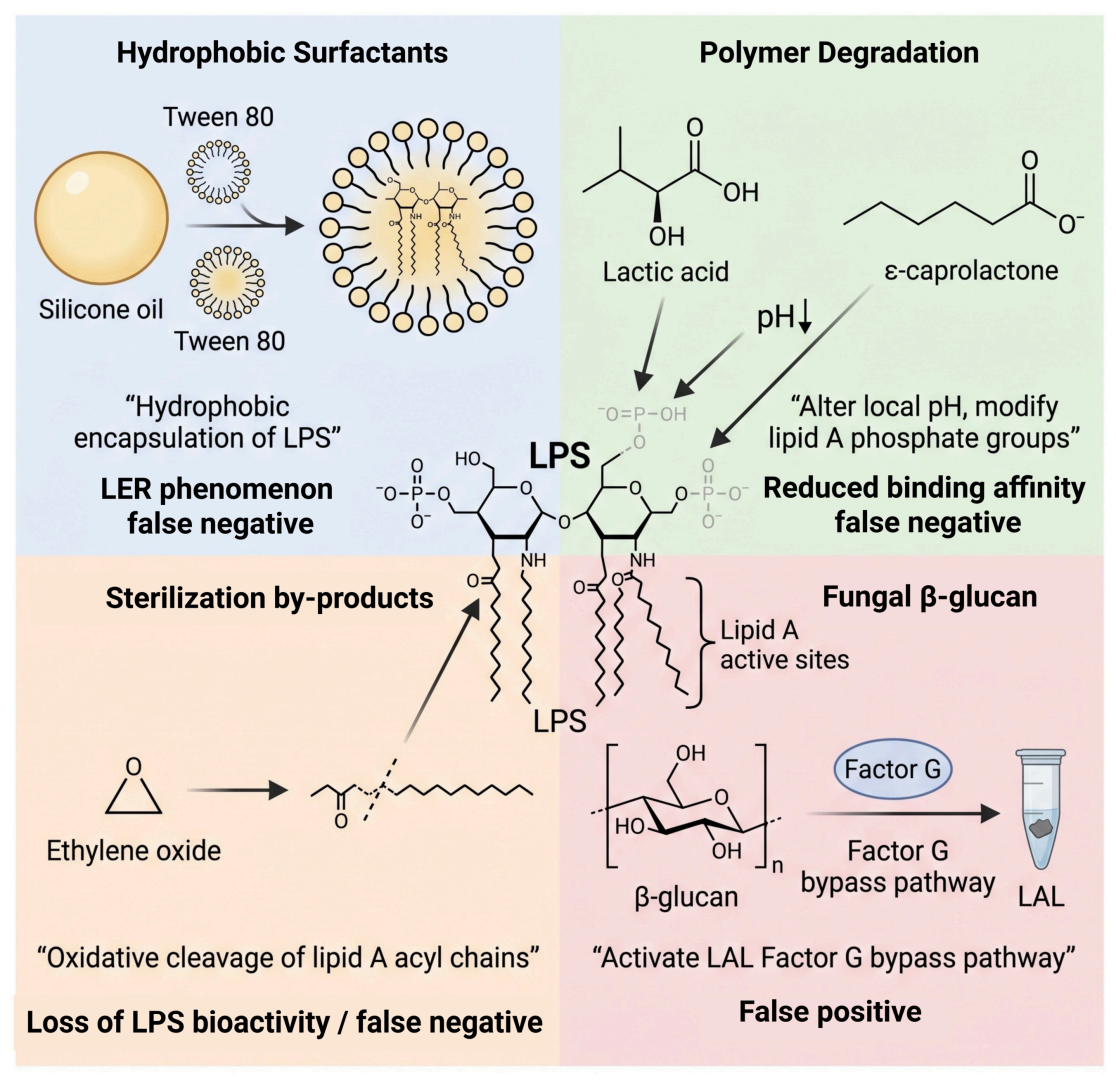
Summarizes four common matrix interference mechanisms in medical device extracts and their effects on detection results. Hydrophobic surfactants (silicone oil, Tween 80) encapsulate LPS within micellar or oil-phase pseudoparticles, shielding lipid A active sites and triggering the Low Endotoxin Recovery (LER) phenomenon, which yields false-negative results. Polymer degradation products (lactic acid, ε-caprolactone) alter local pH and modify the phosphate groups of lipid A, reducing the binding affinity of recognition elements and causing false negatives. Sterilization by-products (residual ethylene oxide) induce oxidative cleavage of lipid A acyl chains, abolishing LPS bioactivity and producing false-negative signals. Fungal (1→3)-β-D-glucan activates the Factor G bypass pathway in the traditional LAL assay, leading to false-positive detection independent of actual endotoxin contamination.

Meanwhile, LAL detection is susceptible to interference from various substances in samples, leading to false-positive or false-negative results. (1→3)-β-D-glucan in samples can activate the Factor G pathway of LAL and interfere with the specific detection of endotoxins [44,45]. Non-ionic surfactants such as Tween-20 and Triton X-100 can form micelles that encapsulate LPS, while buffer components like sodium citrate alter the local pH environment, both contributing to the LER phenomenon [39]. In clinical samples such as human plasma, endotoxins bind to plasma proteins (e.g., lysozyme) to form complexes, which also significantly reduces the recovery rate of LAL detection and compromises the accuracy of results [46]. In addition, traditional methods suffer from insufficient scenario adaptability: the long detection cycles of LAL and rFC assays cannot meet the minute-scale rapid release requirements for online Clean-in-Place (CIP) validation in medical device production lines; the numerous manual operation steps make full-process automation difficult, failing to comply with the data traceability requirements of the medical device GMP system; and the live enzyme systems are susceptible to temperature and pH variations, making them unsuitable for detection needs in non-laboratory environments such as clinical point-of-care settings and manufacturing workshops [47]. Figure 1 summarizes four common matrix interference mechanisms in medical device extracts and their effects on detection results.

## 3. PRR-Based LPS Biosensors: Scenario Applications of Natural Biomimetic Recognition

### 3.1. Toll-like Receptor 4 (TLR4): A Natural High-Affinity Recognition Element

TLR4 is the primary pattern recognition receptor (PRR) in the mammalian innate immune system dedicated to LPS recognition [48]. The heterodimeric complex formed with its essential accessory protein myeloid differentiation factor 2 (MD-2) possesses intrinsically high affinity and specificity for the lipid A moiety of LPS [49], constituting the host’s first line of immune defense against Gram-negative bacterial infections. Binding of LPS to the TLR4/MD-2 complex initiates a conserved downstream signaling cascade that culminates in the secretion of pro-inflammatory cytokines including tumor necrosis factor-α (TNF-α), interleukin-1β (IL-1β), and interleukin-6 (IL-6) [50,51]. This evolutionarily conserved physiological recognition mechanism provides an ideal biomimetic foundation for the development of next-generation endotoxin biosensors.

Biosensors employing recombinant TLR4 protein faithfully recapitulate the natural LPS recognition process, capture target molecules with high precision and fidelity, circumvent the cross-reactivity limitations inherent to traditional antibodies, and significantly enhance detection specificity [52]. In silico molecular docking studies have confirmed that while polycyclic aromatic hydrocarbons (PAHs) and volatile organic compounds (VOCs) exhibit weak binding to TLR4, their binding affinities are 2–3 orders of magnitude lower than that of LPS, providing direct computational evidence for the exceptional specificity of TLR4-mediated LPS recognition [53]. Immobilization of recombinant TLR4 on gold electrodes modified with mixed functionalized self-assembled monolayers (SAMs) enables label-free direct electrochemical detection, offering a robust strategy for the development of rapid, ultrasensitive LPS detection platforms [52]. Furthermore, the structural and functional elucidation of interactions between TLR4 and TIR domain-containing adaptor proteins (e.g., myeloid differentiation primary response 88, MyD88) provides a solid theoretical framework for optimizing sensor signal transduction efficiency [54]. Collectively, as a natural high-affinity and high-specificity biorecognition element, TLR4 holds significant promise for addressing the unmet needs of endotoxin detection in medical device manufacturing, online cleaning validation, and clinical point-of-care settings.

### 3.2. Interface Optimization and Performance Enhancement of TLR4 Biosensors

**Figure 2:**
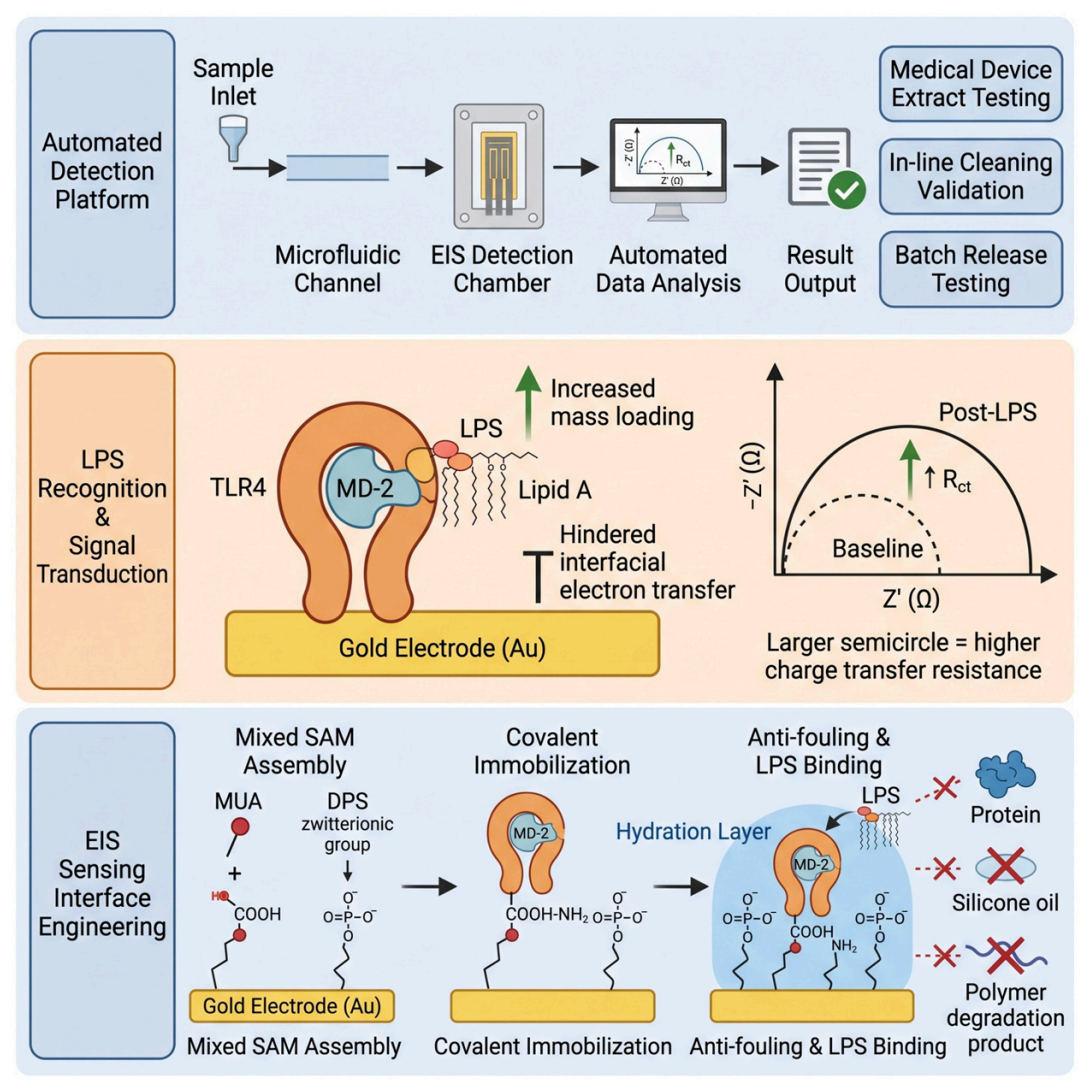
Schematic of the TLR4/MD-2-based EIS biosensor for endotoxin detection. The top panel shows the automated detection workflow: sample injection through a microfluidic channel, EIS measurement in the detection chamber, automated data analysis, and result output—supporting medical device extract testing, in-line cleaning validation, and batch release. The middle panel illustrates the sensing mechanism: LPS binding to the TLR4/MD-2 complex immobilized on the gold electrode increases mass loading and hinders interfacial electron transfer, raising the charge transfer resistance (R_ct_). The bottom panel details the MUA-DPS mixed self-assembled monolayer (SAM) interface engineering, which enables oriented covalent immobilization of MD-2 while forming a hydration layer that repels non-specific adsorption of proteins, silicone oil, and polymer degradation products.

The core challenge in constructing TLR4-based biosensors lies in achieving efficient and oriented immobilization of TLR4 proteins on the sensing interface, while simultaneously minimizing non-specific adsorption of proteins and hydrophobic substances present in medical device extracts, thereby ensuring reliable detection in complex matrices. Potential-assisted self-assembly (PASA) technology has emerged as a transformative breakthrough in biosensor interface engineering: a mixed solution of 11-mercaptoundecanoic acid (MUA) and zwitterionic 3-(dimethyl(3-sulfopropyl) ammonium) propanethiol (DPS) can be rapidly assembled onto gold electrode surfaces within 5 min, reducing assembly time by over 200-fold compared to conventional overnight self-assembly, and laying a critical foundation for scalable manufacturing of sensor chips [52]. The MUA-DPS mixed SAM fabricated via this method exhibits excellent antifouling performance, effectively resisting non-specific protein adsorption from complex matrices including human plasma and medical device extracts, thereby preventing false-positive and false-negative results caused by matrix interference and ensuring detection accuracy in practical applications. Additionally, the terminal carboxyl groups of MUA provide reactive sites for covalent immobilization of TLR4 proteins, enabling oriented immobilization that preserves the native conformation and ligand-binding activity of the receptor. The EIS sensor based on the TLR4/MUA-DPS/Au architecture exhibits an ultralow limit of detection (LOD) of 4 ng/mL for LPS derived from Escherichia coli O157:H7, with a broad linear detection range of 1–1000 ng/mL. It shows no significant matrix interference in spiked plasma samples, confirming its strong anti-interference capability and practical application reliability [46]. Figure 2 shows the overall design of the TLR4-based EIS biosensor and its antifouling interface engineering strategy.

### 3.3. Medical Device Scenario Applicability and Technical Limitations

TLR4 biosensors have significant application advantages in medical device quality control scenarios: First, their inherent high specificity can avoid interference from β-glucan, making them suitable for the detection of special samples such as interventional devices with glucan coatings and absorbable sutures [52]. Second, the zwitterionic antifouling interface has the potential to mitigate LER caused by hydrophobic interferents in medical device extracts. Third, their label-free and rapid detection characteristics can meet the rapid release requirements for online cleaning verification in production lines [50]. Nevertheless, three key bottlenecks currently restrict the industrial translation and large-scale application of this technology. First, the production cost of recombinant TLR4/MD-2 complexes remains prohibitive, and there is significant batch-to-batch variability in protein activity persists, hindering the realization of large-scale standardized production [55]. Second, protein-based recognition elements have poor storage stability, are prone to inactivation at room temperature, and have a short shelf life, which cannot meet the requirements for large-volume and long-term storage in industrial quality control laboratories [56]. Third, long-term exposure of proteins to complex medical device extracts is prone to denaturation, leading to sensor baseline drift and reduced detection reproducibility.

These technical and economic bottlenecks constitute the primary barriers to widespread industrial adoption of TLR4 biosensors. Addressing these challenges will require coordinated breakthroughs in recombinant protein manufacturing, stabilization strategies, and interfacial design.

## 4. Genetically Engineered Recognition Element-Based Sensors: Controllable Standardized Solutions

### 4.1. Genetically Engineered Recognition Elements: Overcoming Antibody Heterogeneity Bottlenecks

LPS consists of three components: O-antigen, core polysaccharide, and lipid A. Among them, the structure of O-antigen exhibits high strain heterogeneity, while lipid A is the core active conserved epitope of endotoxins. Conventional polyclonal antibodies predominantly target the variable O-antigen region, while most monoclonal antibodies also exhibit strain-dependent affinity variations due to epitope heterogeneity [57]. This leads to problems such as cross-reactivity and unstable detection results, making it difficult to meet the standardized requirements of medical device quality control. Genetic engineering antibody technology enables precise and targeted modification of antibody variable regions, allowing the screening of high-affinity single-chain variable fragments (scFv) targeting the conserved epitope of LPS lipid A from large-capacity antibody libraries. Such recognition elements can achieve broad-spectrum recognition of LPS from different strains, and their batch-to-batch consistency is far superior to that of traditional antibodies, enabling standardized and large-scale production [57]. Innovative studies have constructed a fusion protein of CD14 (a natural LPS-binding protein) and the IgE Fcεfragment (CD14-FcεIgE). Leveraging the modular design of genetically engineered antibodies, it uses CD14 to achieve high-affinity binding to LPS and the Fcεfragment to precisely anchor to the FcεRI receptors on the surface of mast cells (RBL-2H3 cell line). This converts the LPS recognition event from solution-phase binding to specific cross-linking on the cell membrane, laying a foundation for subsequent signal transduction and amplification [58].

### 4.2. Genetically Engineered Element-Mediated Microfluidic Cell Sensors

**Figure 3:**
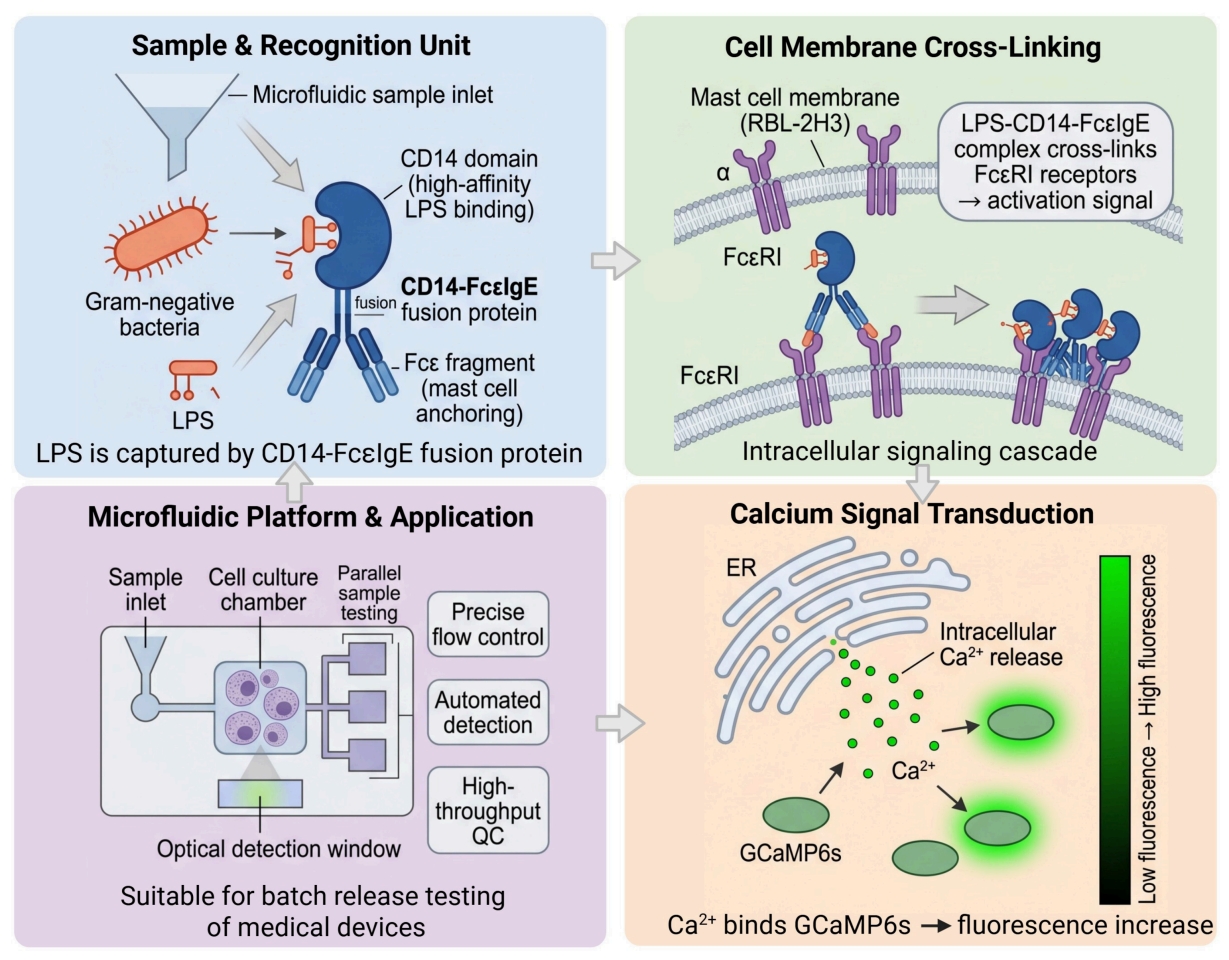
Schematic illustration of the CD14-FcεIgE-based microfluidic cell biosensor for LPS detection. The system comprises four interlinked functional modules. (i) Sample and recognition unit: LPS from Gram-negative bacteria is captured with high affinity by the CD14 domain of the CD14-FcεIgE fusion protein. (ii) Cell membrane cross-linking: The LPS-bound fusion protein cross-links FcεRI receptors on the surface of RBL-2H3 mast cells, initiating intracellular signaling cascades. (iii) Calcium signal transduction: Receptor activation triggers Ca^2+^ release from endoplasmic reticulum stores; the calcium indicator GCaMP6s binds free Ca^2+^ and generates a quantifiable fluorescence increase. (iv) Microfluidic platform: Integrated chip design enables precise flow control, parallel sample processing, and automated optical detection, supporting high-throughput batch release testing of medical devices.

The advantages of genetically engineered recognition elements have been fully unleashed on microfluidic cell sensing platforms. This platform achieves deep integration of biological recognition, cellular signal transduction, and miniaturized fluid control technologies, and can meet the high-throughput and automated quality control requirements of medical devices. The core of the system constructed by JIANG H et al. [58] is the RBL-2H3 mast cell line stably expressing the calcium indicator protein GCaMP6s. When LPS or Gram-negative bacteria in samples are captured by CD14-FcεIgE and cross-link the FcεRI receptors on the cell surface, the mast cell activation pathway is triggered. Intracellular calcium stores release Ca^2+^, and the sudden increase in Ca^2+^ concentration is monitored in real time by GCaMP6s. The change in fluorescence intensity directly reflects the presence of the target analyte, achieving efficient conversion of chemical recognition into optical signals. GCaMP6s operates via an allosteric photophysical mechanism: Ca^2+^ binding to calmodulin triggers a conformational rearrangement that restores the intact β-barrel of the circularly permuted GFP chromophore, sharply increasing fluorescence quantum yield [59]. This calcium-dependent optical switching converts intracellular signaling events directly into measurable fluorescence intensity. Through precision microchannels and reaction chambers, microfluidic chips can precisely regulate the cell culture environment, sample injection flow rate, and contact time between cells and target analytes, ensuring detection consistency and reproducibility [60]. Meanwhile, they support the parallel integration of multiple detection units to realize simultaneous detection of multiple samples, significantly improving screening throughput. Figure 3 illustrates the working principle of the CD14-FcεIgE-based microfluidic cell biosensor for LPS detection.

### 4.3. Advantages and Limitations for Medical Device QC

This technology offers distinct advantages for endotoxin quality control in medical device manufacturing. One core advantage lies in genetically engineered recognition elements, which support standardized, scalable production with minimal batch-to-batch variability, aligning with the stringent reproducibility requirements of medical device quality control under GMP frameworks. Furthermore, the integrated microfluidic platform enables high-throughput detection with reduced reagent consumption, making it well suited for batch release testing of finished medical devices. Importantly, the cell activation-based detection modality directly reflects the mammalian pro-inflammatory toxicity of LPS, providing a more biologically relevant assessment of pyrogenic risk than traditional horseshoe crab enzymatic assays that only measure enzymatic reactivity.

Despite these merits, several limitations remain that hinder widespread industrial deployment of this technology. Most critically, living cell-based sensors rely on stringent culture conditions and cold-chain storage and transport. Their very limited shelf life renders them unsuitable for deployment in non-laboratory settings, including medical device production floors and point-of-care clinical sites [61]. Moreover, cellular detection systems are vulnerable to interfering components in medical device extracts, such as sterilization byproducts and cytotoxic compounds, which can distort cellular physiological responses and introduce systematic bias into detection results. Compounding these technical barriers, the high production cost of genetically engineered recognition elements limits reductions in per-assay testing costs, placing the technology at a competitive disadvantage relative to mature, low-cost LAL assays in the current market.

## 5. EIS Sensing Technology: Core Carrier for Automated Medical Device Detection

### 5.1. Principles and Core Advantages of EIS Technology

EIS is a label-free, highly sensitive electrochemical technique that can be readily miniaturized, serving as the core technical platform for developing automated endotoxin detection systems for medical devices. Its detection principle relies on real-time monitoring of biological recognition events by measuring changes in charge transfer resistance (R_ct_) at the electrode–electrolyte interface [62]. Physically, when LPS binds to the specific recognition elements immobilized on the electrode surface, it forms an insulating biomolecular layer that impedes the diffusion of redox probes and raises R_ct_ proportionally with analyte concentration. Because R_ct_ is extremely sensitive to variations in interfacial mass loading and surface properties, the magnitude of this change exhibits a strong positive correlation with LPS concentration, enabling accurate quantitative detection of the target analyte [63].

### 5.2. Sensor Interface Engineering: Antifouling Design and Matrix Compatibility

Biofouling and matrix interference represent major barriers to the reliable application of EIS sensors in complex medical device extracts. Non-specific adsorption of sample components onto the electrode surface induces baseline drift and signal distortion, severely compromising detection accuracy and reproducibility. Among existing interfacial modification strategies, zwitterionic mixed SAMs are widely recognized as one of the most promising approaches for improving antifouling performance and matrix compatibility [52].

The MUA-DPS zwitterionic hybrid interface fabricated via potential-assisted self-assembly can form a dense hydration layer on the electrode surface. It strongly inhibits the non-specific adsorption of proteins, silicone oil and polymer degradation products present in medical device extracts [52]. Meanwhile, it provides active sites for the oriented immobilization of recognition elements such as TLR4 and aptamers, achieving a good balance between antifouling capability and recognition activity [64]. Such interface engineering not only guarantees high selectivity of sensors in complex environments but also enhances long-term stability, laying a solid foundation for the development of reusable and disposable automated detection chips [65]. In addition, photocatalytic regeneration and peptide re-immobilization techniques allow repeated cycling of electrodes with negligible performance deviation, further improving the practicality of the sensors [63]. Innovations in antifouling design and stability are critical for translating laboratory-based LPS detection technologies to on-site regulatory testing and clinical applications.

## 6. System Integration & Intelligent Analysis for Full-Process Medical Device QC

### 6.1. Integration of Microfluidic and Sensing Modules

The core development direction of endotoxin detection for medical devices in the future is to realize “sample-in, answer-out” full-process automated detection [66]. The key is to integrate functional modules including sample introduction, pretreatment, reaction, sensing detection, waste disposal, and data output into a single microfluidic chip or compact device, adapting to the quality control requirements throughout the entire life cycle of medical devices [67]. Microfluidic technology can precisely manipulate fluids at the micrometer scale and complete reagent mixing and reactions in nanoliter to microliter volumes, significantly reducing the consumption of expensive reagents such as LAL and improving detection throughput [68]. The deep integration of sensing modules and microfluidic processing units is the core pathway for upgrading detection capabilities. Integrating TLR4 receptors and cell sensor units into multi-channel chips enables simultaneous detection of multiple samples or multiple analytes (LPS, bacterial DNA) [69]. For example, a microfluidic platform integrating dielectrophoresis (DEP) and cell tracking can distinguish Escherichia coli (E.coli) with different LPS glycoforms in a high-throughput and non-invasive manner, providing a new method for rapid pathogen typing based on LPS structure [70]. A multifunctional microfluidic chip coupled with perfusion cell culture and SERS detection can realize in situ real-time continuous monitoring of cytokines such as IL-6 [71]. The above technologies fully demonstrate the advantages of seamless integration between biosensing and microfluidic processes, providing a solid foundation for the construction of automated and multifunctional LPS detection systems.

### 6.2. Intelligent Signal Processing and Regulatory-Compliant Data Analysis

The performance of automated endotoxin detection platforms relies on the synergy between precision hardware integration, advanced signal processing, and intelligent data analysis systems [72]. The core of the system consists of high-precision signal acquisition devices (electrochemical workstations, fluorescence microscopes) and real-time data acquisition modules, ensuring complete and accurate capture and digitization of raw detection signals [71]. For instance, impedance-based aptamer sensors enable the quantitative detection of endotoxins via EIS. The continuous flow nature of microfluidic systems facilitates target analyte enrichment, reducing the LOD from 5 ng/mL to 500 pg/mL while shortening the detection time [73].

Machine learning algorithms play a pivotal role in the in-depth analysis and intelligent interpretation of complex signals, enabling automatic identification of characteristic signals, quantification of LPS concentration, and assessment of matrix interference. CHEN H et al. developed a visual detection method for LPS based on hybridization chain reaction (HCR), gold nanoparticle aggregation, and electrophoretic accumulation [72]. It automatically analyzes the integrated density of gold nanoparticle aggregates in microchannels through image analysis algorithms to achieve quantitative detection of LPS. Temporal dynamics data of cytokine secretion by macrophages stimulated with LPS requires efficient analysis using intelligent algorithms [74]. The final detection results are presented via a user-friendly software interface, converting raw EIS spectra, fluorescence kinetic curves, and image data into intuitive “pass/fail” judgments or concentration value reports. The intelligent platform (CryoSIM) integrating microfluidics, deep learning, and transport modeling achieves high-precision cell segmentation through deep learning and calculates key parameters via custom interfaces, significantly improving analysis throughput and reproducibility [75]. The POCT-oriented USENSE device integrates lateral flow microfluidic channels, sensor arrays, and random forest models, enabling detection of biomarkers such as LPS in urine within minutes and completing disease classification and recurrence risk assessment [60]. This marks that endotoxin detection has transitioned from manual interpretation-based endpoint measurement to a new stage of full-process automation, data-driven operation, and intelligent decision-making [76,77].

### 6.3. Comprehensive Performance Comparison of Various Endotoxin Detection Technologies

**Figure 4:**
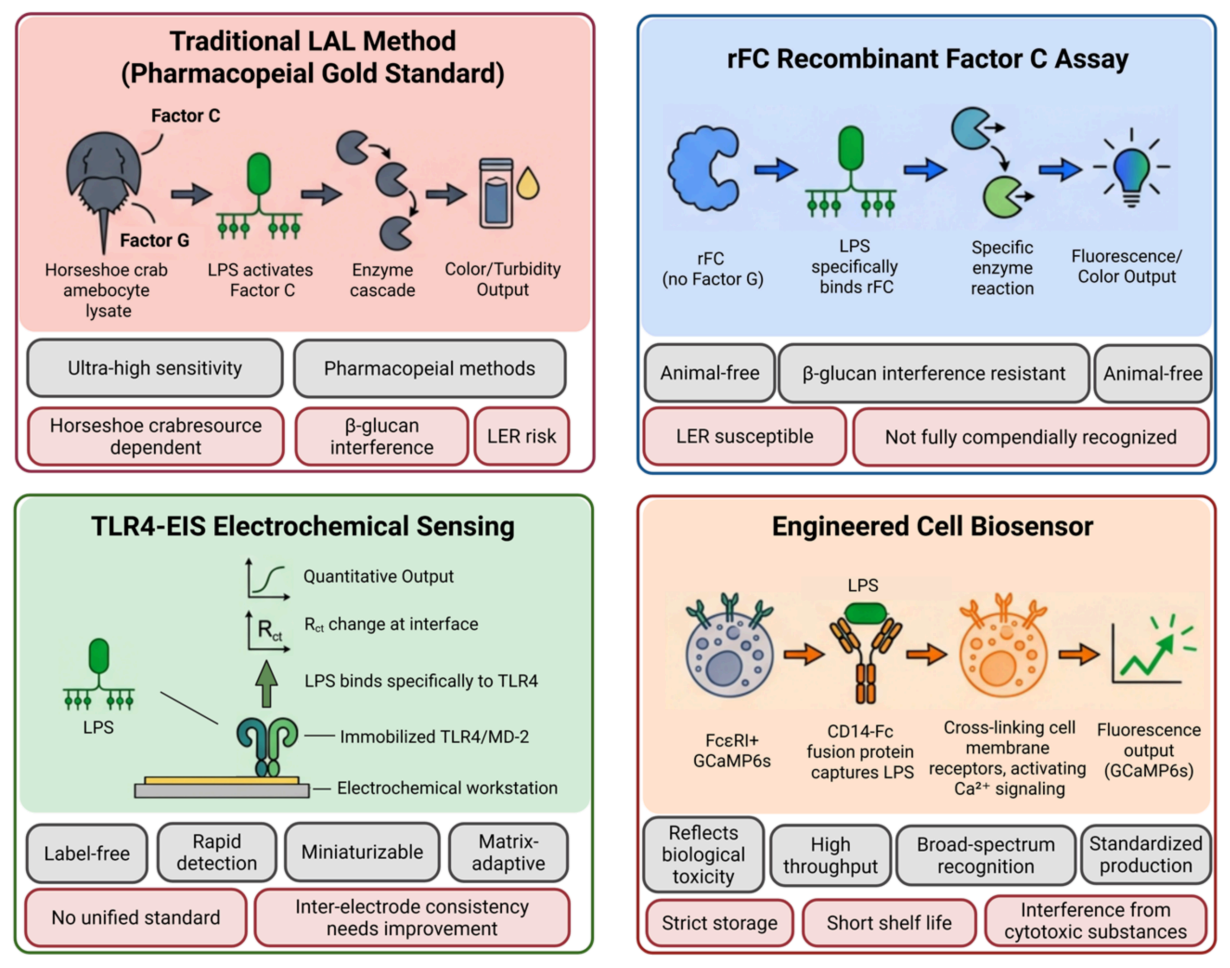
Schematic comparison of four core endotoxin detection technologies: mechanisms and feature overview. Each panel illustrates the recognition mechanism, signal transduction route, and key advantages/limitations of the traditional LAL assay, rFC-based assay, TLR4-EIS electrochemical sensing, and engineered cell biosensor for reference in medical device technology selection.

To provide an intuitive, side-by-side overview of the technical characteristics of different detection systems, Figure 4 visually summarizes the core reaction pathways, key strengths, and major translational limitations of four mainstream endotoxin detection technologies for medical device quality control.

To clearly demonstrate the applicability of various emerging technologies in medical device scenarios, this paper conducts a horizontal comparison of different technologies from the dimensions of core performance, scenario applicability, and industrialization maturity. LAL and rFC assays offer the highest sensitivity (0.001–0.005 EU/mL) and full pharmacopoeial recognition, making them the gold standard for compliance testing, but they suffer from poor matrix tolerance and low automation. TLR4-EIS biosensors excel in anti-interference and detection speed (15–30 min) with full microfluidic automation potential, yet lack unified standards and remain at the prototype stage. Engineered cell biosensors provide biologically relevant pyrogenicity assessment and high throughput, but require strict cold-chain storage and are vulnerable to cytotoxic matrix components. The results are presented in Table 3.

## 7. Discussion

Although novel LPS-based sensor technologies have demonstrated enormous potential in the field of endotoxin detection for medical devices, the path from laboratory prototypes to regulatory-approved industrial products still faces a series of formidable challenges. The primary challenge lies in the lack of standardization and validation. Currently, the LAL assay relies on well-established international standards such as USP and EP to become the industry gold standard [12], while novel sensors based on electrochemistry, SERS, and fluorescence lack unified international standards. Extensive and rigorous comparative studies are required to accumulate sufficient validation data and demonstrate their equivalence or superiority to the LAL assay in terms of sensitivity, specificity, precision, and accuracy before they can obtain regulatory approval [89]. Notably, the US Pharmacopeia officially incorporated the rFC assay into General Chapter <85> as a compendial method, with the 2025 revision further refining its application scope for medical devices, marking a major milestone in global regulatory harmonization. However, disparities remain in the adoption timeline across different regions, with some pharmacopoeias still in the process of updating their standards [90]. In addition, the issue of universality for complex samples urgently needs to be addressed. Medical device extracts have complex matrices containing interferents such as surfactants, metal ions, drug residues, and proteins. These interferents tend to mask endotoxins through electrostatic interactions, hydrophobic interactions, or complex formation, triggering the LER phenomenon and resulting in false negatives [91,92]. Structural variations in LPS and interactions with plasma proteins constitute multiple masking mechanisms [45]. Apart from matrix masking effects, LPS aggregation is another easily ignored factor that disturbs detection accuracy [93]. As amphipathic molecules, LPS self-assembles into micelles in water, and the aggregation level determines how readily lipid A can bind to recognition receptors. The analysis of Hernández-Acosta et al. on nanoparticle aggregation and separation dynamics (including sedimentation and Brownian motion) provides a physical basis to explain how aggregate size and structure alter binding kinetics and assay sensitivity [94]. Aggregated LPS presents fewer accessible lipid A epitopes per unit mass than dispersed monomers, which contributes to the LER effect and can shift the linear detection range of biosensors. Strategies to control LPS dispersion through optimized sample pretreatment may therefore serve as a complementary approach to improving detection reliability. Novel sensors need to be validated for robustness in extremely complex matrices to achieve interference-resistant and accurate recovery detection [95]. Finally, long-term stability and cost control are the key bottlenecks for industrialization. Biological recognition elements such as TLR4 proteins, engineered cells, and specific aptamers are prone to inactivation during storage and transportation, limiting the shelf life and batch-to-batch consistency of sensors [96]. Sensor chip fabrication, biological element immobilization processes, and ensuring uniformity in large-scale production all face cost control challenges. It is necessary to reduce the per-test cost and extend product shelf life while maintaining high performance to achieve the leap from “laboratory prototypes” to “industrialized products”.

Looking ahead, LPS-based endotoxin detection technologies for medical devices will develop in the directions of multimodality, intelligence, integration, and portability. Multimodal and multiplex detection stands as a major developmental trend for endotoxin and pyrogen testing. Traditional LAL assays are primarily designed for the detection of endotoxins, yet medical devices are also susceptible to contamination by other pyrogenic contaminants, including peptidoglycan from Gram-positive bacteria and fungal β-glucan [97]. Accordingly, future research efforts are increasingly directed toward the development of “all-in-one” integrated sensor platforms capable of simultaneous detection of multiple pyrogenic species. For instance, assays based on the monocyte activation test (MAT) principle can respond to a broad spectrum of Toll-like receptor agonists including LPS by measuring the release of pro-inflammatory cytokines such as IL-6, thereby delivering a more comprehensive assessment of bioburden and pyrogenic risk [96,98]. This multi-parameter detection capacity is particularly valuable for safety evaluation of complex medical devices, such as products incorporating bio-derived materials. Alongside advances in multiplexed detection, point-of-care testing (POCT) and online process monitoring constitute another pivotal direction for accelerating the translational adoption of these technologies. Current endotoxin detection practices remain largely confined to central laboratory settings, characterized by labor-intensive protocols and prolonged turnaround times. Moving forward, research priorities will center on advancing the miniaturization and portability of detection systems, with the ultimate goal of enabling real-time, on-site endotoxin monitoring across production lines and clinical point-of-care settings such as dialysis centers and operating rooms [99]. As a representative example, field-effect transistor (FET)-based aptamer sensors have been reported to enable rapid detection of endotoxins and intact Escherichia coli cells in human serum within 300 s, highlighting substantial promise for POCT applications [99]. Furthermore, the inherent automation and parallel processing capabilities of microfluidic technology can substantially reduce reagent consumption and minimize human-induced error, offering a viable technical pathway for high-throughput, miniaturized POCT platforms [22]. Beyond detection of free LPS molecules, emerging strategies are extending endotoxin measurement to LPS-bearing biological nanoparticles. For instance, nanoflow cytometry coupled with immunolabeling enables quantitative profiling of LPS-positive outer membrane vesicles (OMVs) in biological fluids, offering a new dimension for characterizing Gram-negative bacterial contamination [100]. This vesicle-targeted detection paradigm, while currently applied primarily in clinical diagnostics, may find future utility in medical device quality control for assessing viable but non-culturable bacterial residues and low-level contamination that conventional assays may overlook.

**Figure 5:**
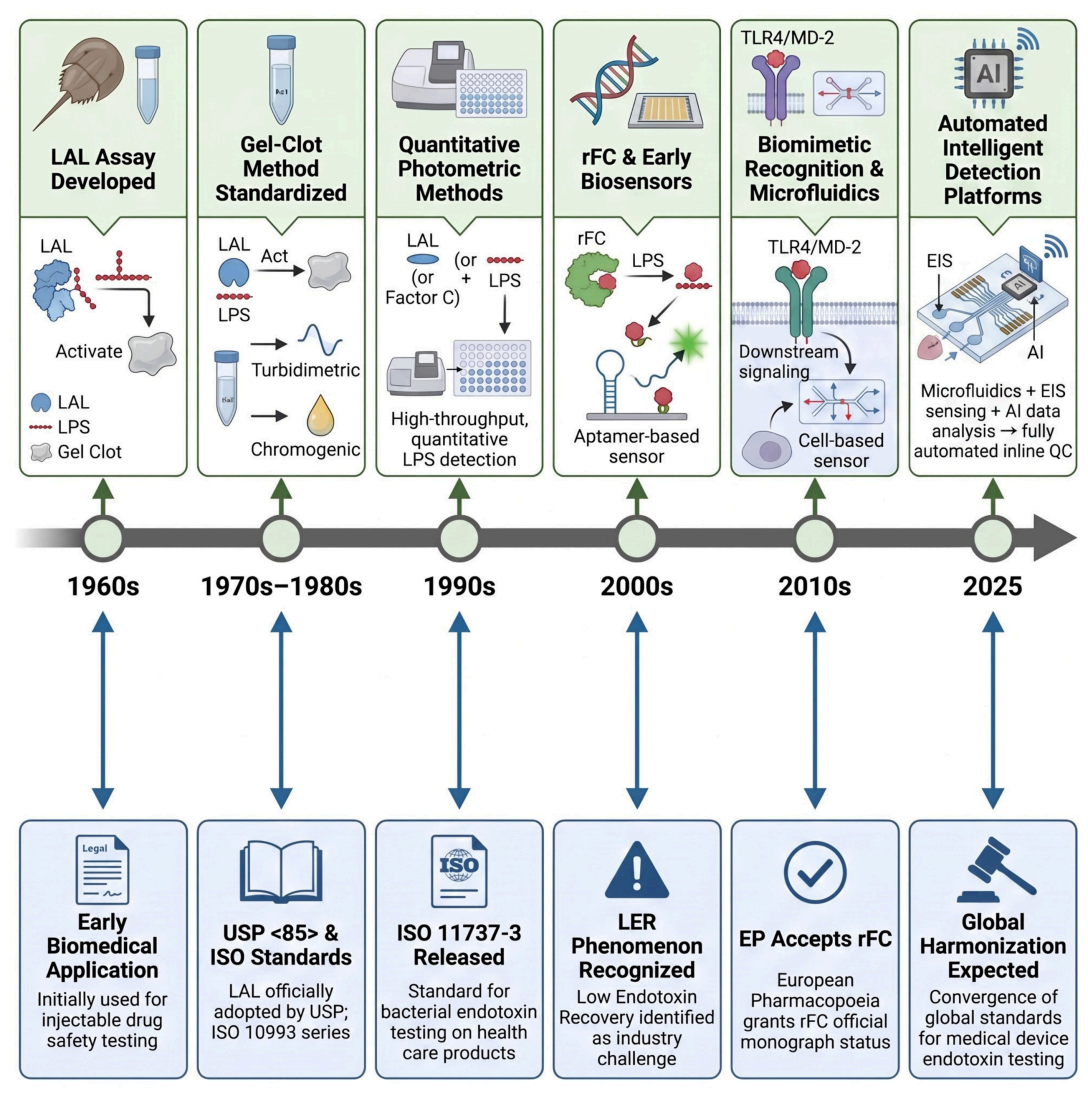
Historical evolution and developmental timeline of endotoxin detection technologies. The upper trajectory traces the technological progression from the original Limulus Amebocyte Lysate (LAL) assay, through standardized gel-clot and quantitative photometric methods, recombinant Factor C (rFC) biosensors, and TLR4-mediated biomimetic recognition integrated with microfluidics, toward next-generation automated intelligent detection platforms. The lower timeline correlates key regulatory and standardization milestones—including early biomedical adoption, USP <85>/ISO harmonization, the recognition of the Low Endotoxin Recovery (LER) phenomenon, European Pharmacopoeia acceptance of rFC—and the anticipated trajectory toward global regulatory convergence for medical device endotoxin testing.

Furthermore, artificial intelligence (AI) will deeply empower sensor design. Computational models (e.g., molecular docking) can predict and optimize the interactions between recognition elements (e.g., aptamers, TLR4/MD-2 complexes) and LPS [101], enabling the rational design of synthetic receptors with higher affinity or novel specificities [102]. Meanwhile, AI and machine learning algorithms (e.g., Support Vector Machines, deep learning network RamanNet) can be applied to the analysis of complex spectral data (e.g., Surface-Enhanced Raman Spectroscopy) to achieve accurate classification and identification of endotoxins from different sources, enhancing the intelligence level of detection [103,104]. Finally, deep integration with intelligent manufacturing will be the ultimate goal. Automated and miniaturized endotoxin detection modules will be seamlessly integrated into the cleaning, disinfection, or packaging production lines of medical devices, enabling closed-loop management of quality control and real-time release testing [22]. This “on-line” quality assurance system can promptly identify contamination risks during the production process and ensure the safety of medical devices at the source, aligning with the development concepts of Industry and intelligent pharmaceutical manufacturing. Figure 5 outlines the six-decade evolution of endotoxin detection technologies and the corresponding regulatory milestones.

Through the synergistic development of the above directions, future endotoxin detection technologies will be more sensitive, rapid, comprehensive, and automated, safeguarding the safety of medical devices and pharmaceuticals.

## 8. Conclusions

The core challenges of endotoxin detection for medical devices have evolved beyond the traditional bottleneck of insufficient sensitivity, and now center on three key demands: result reliability in complex matrices, compatibility with automated workflows, and alignment with regulatory compliance requirements. TLR4 pattern recognition receptors and genetically engineered recognition elements effectively mitigate the limitations of traditional methods—including insufficient specificity and susceptibility to false positives—at the biological recognition level. Meanwhile, EIS electrochemical sensing and microfluidic integration technologies provide robust engineering support for automated, miniaturized and intelligent detection workflows, offering diversified technical solutions for quality control across the entire medical device lifecycle.

A key research gap in the current field lies in the deep adaptation of emerging technologies to medical device-specific scenarios, and their industrial translation from laboratory prototypes to regulatory-approved products. Future research should prioritize three major directions: the development of highly stable synthetic recognition elements to address the storage stability and cost bottlenecks of biological recognition components; the optimization of anti-interference technologies for complex matrices to systematically improve LER caused by medical device-specific matrices; and the standardization of detection methodologies alongside regulatory equivalence validation to facilitate global regulatory recognition of novel technologies. Breaking through the barriers between laboratory innovation and industrial deployment will enable the widespread implementation of highly automated, intelligent and regulatory-compliant endotoxin detection platforms, strengthening biosafety safeguards for medical devices at the source and protecting the health and safety of clinical patients.

## Figures and Tables

**Table 1:** Core Requirements of Major Global Regulatory Standards for Endotoxin Testing in Medical Devices.

Standard Number	Core Scope of Application	Mandatory Requirements	Refs.
ISO 10993-12: 2021	All medical devices contacting the human body	Specifies extraction methods (temperature, time, medium), principles for setting endotoxin limits, and requirements for methodological validation based on contact type.	[6]
GB/T 14233.2-2005	Medical infusion, transfusion, and injection devices in China	Clarifies the implementation standards for finished product endotoxin limits, detection methods, and interference tests	[7]
USP <85>	Medical devices and pharmaceuticals marketed in the United States	Specifies the methodological validation indicators (recovery rate, precision, specificity) for endotoxin testing, and clarifies the control requirements for the LER phenomenon	[8,30]
EP 2.6.14	Medical devices and pharmaceuticals marketed in the European Union	Recognizes the legal status of the recombinant Factor C (rFC) assay, and specifies the interference test procedures for complex matrix samples	[9]

**Table 2:** Common Interferents and Action Mechanisms for Endotoxin Testing in Medical Device Extracts.

Interfering Substance	Representative Substances	Mechanism	Impact Detection Methods	Refs.
Hydrophobic Surfactants	Silicone oil, Polysorbate 80	Encapsulate LPS to form oil-phase “pseudoparticles”, shield the active sites of lipid A, and trigger the LER phenomenon	LAL assay, rFC assay, TLR4-EIS biosensing	[29,36,37,38]
Polymer Degradation Products	Lactic acid, ε-caprolactone	Alter local pH, modify the phosphate groups of LPS lipid A, and reduce its binding affinity to recognition elements	Antibody-based biosensors, Surface-Enhanced Raman Scattering (SERS) biosensing	[39,40,41]
Sterilization By-products	Residual ethylene oxide, 2-chloroethanol	Oxidatively modify the lipid A moiety of LPS and destroy its bioactive epitopes	LAL assay, rFC assay, aptamer-based biosensors	[42]
Fungal Contaminants	(1→3)-β-D-glucan	Activate the Factor G bypass pathway of the LAL assay, leading to false-positive results	Traditional LAL assay	[43]

**Table 3:** Performance Comparison of Endotoxin Detection Technologies in Medical Devices.

Type	Core Recognition Element	LOD	Detection Time	Anti-Matrix Interference Capability	Automation Level	Regulatory Compliance	Industrial Maturity	Core Application Scenarios	Refs.
Traditional LAL Assay	Limulus amebocyte lysate (multi-factor coagulation cascade)	0.005 EU/mL	1~4 h	Poor	Low	Recognized by Global Pharmacopoeias	Fully Commercialized	Laboratory Finished Product Compliance Testing	[78,79]
rFC Assay	Recombinant Factor C	0.001~ 5 EU/mL	1~2 h	Moderate	Medium	Recognized by Partial Pharmacopoeias	Fully Commercialized	Laboratory Finished Product Compliance Testing	[80,81,82]
TLR4-EIS Sensing	TLR4/MD-2 Complex	4 ng/mL	15~30 min	Good	High	No Unified Standards	Laboratory Prototype/Small-Scale Commercialization	On-Line Production Line Verification, On-Site Rapid Screening	[83,84,85]
Genetically engineered cell-based biosensing	Genetically Engineered Fusion Protein	80 CFU /mL	10~30 min	Moderate	High	No Unified Standards	Laboratory Prototype	Laboratory High-Throughput Screening	[58,86]
Microfluidics-integrated EIS biosensing	Aptamers/Antimicrobial Peptides	0.5 pg /mL	20~30 min	Good	Very High	No Unified Standards	Partially Commercialized	Full-Process Automated Quality Control, On-Site Rapid Detection	[73,87,88]

## Data Availability

No new data were created or analyzed in this study. Data sharing is not applicable to this article.

## Abbreviations

The following abbreviations are used in this manuscript:

LPS	Lipopolysaccharide
TLR4	Toll-like receptor 4
EIS	Electrochemical Impedance Spectroscopy
LAL	Limulus Amebocyte Lysate
pg	picogram
LER	Low Endotoxin Recovery
rFC	recombinant Factor C
DEHP	Di-2-ethylhexyl phthalate
SERS	Surface-Enhanced Raman Scattering
CIP	Clean-in-Place
PRR	pattern recognition receptor
MD-2	myeloid differentiation factor 2
TNF-α	tumor necrosis factor-α
IL-1β	interleukin-1β
IL-6	interleukin-6
PAHs	polycyclic aromatic hydrocarbons
VOCs	volatile organic compounds
SAMs	self-assembled monolayers
PASA	Potential-assisted self-assembly
LOD	limit of detection
MUA	mercaptoundecanoic acid
scFv	single-chain variable fragments
ALP	alkaline phosphatase
HCR	hybridization chain reaction
POCT	point-of-care testing
FET	field-effect transistor
AI	artificial intelligence
